# Pea Seedling Histaminase as a Novel Therapeutic Approach to Anaphylactic and Inflammatory Disorders

**DOI:** 10.1100/tsw.2007.139

**Published:** 2007-06-12

**Authors:** Emanuela Masini, Danielle Bani, Cosimo Marzocca, Mircea Alexandru Mateescu, Pier Francesco Mannaioni, Rodolfo Federico, Bruno Mondovì

**Affiliations:** ^1^ Department of Preclinical and Clinical Pharmacology, University of Florence, Italy; ^2^ Department of Anatomy, Histology and Forensic Medicine, Section of Histology, University of Florence, Italy; ^3^ Department of Chemistry and Biochemistry, Université du Québec à Montréal, Montreal (Quebec), Canada; ^4^ Department of Biology, University of Rome, Italy; ^5^ Department of Biochemical Sciences “A. Rossi Fanelli”, Rome University “La Sapienza”, Rome, Italy

**Keywords:** pea seedling histaminase, copper amine oxidases, inflammation, cardiac anaphylaxis, asthma-like reaction, cardiac ischemia-reperfusion, splanchnic circulation occlusion and reperfusion

## Abstract

Amine oxidases (AOs) are ubiquitous enzymes involved in the metabolism of biogenic amines. Copper AOs (Cu-AOs) catalyze the oxidative deamination of primary amine groups of several biogenic amines, such as putrescine, cadaverine, and histamine. In the present review, the effects of a plant amine oxidase (Cu-AO, histaminase, EC1.4.3.6) purified from pea seedlings in the modulation of IgE-mediated allergic reactions, and in the prevention of cardiac and splachnic postischemic reperfusion damage are reported.

## OVERVIEW

### Biochemistry of Amine Oxidases

Amine oxidases (AOs) are a broad class of Cu- or FAD-containing enzymes (Cu- and FAD-AOs) distributed among all living organisms, which are able to oxidize biogenic amines to hydrogen peroxide ($H_2O_2$) and aldehydes. Their widespread occurrence accounts for an undoubtedly relevant function in biogenic amine metabolism. AOs are heterogeneous in structure, catalytic mechanisms, and mode of substrate oxidation. Mono, di-, and polyamines are oxidatively deaminated by AOs in a reaction consuming $O_2$ and $H_2O$, and producing the corresponding aldehyde, the removed amine moiety, and $H_2O_2$ in stoichometric amounts according to the following reaction:

$$H_2NH_2 + O_2 + H_2O \quad \rightarrow \quad RCHO + NH_3 + H_2O_2$$

Cu-AO contains a second cofactor, topaquinone (TPQ)[1], which is produced by post-translational oxidation of a tyrosine residue catalyzed by the same $Cu^{2+}$[2,3]. The second group of AOs contains FAD as a cofactor and includes cytosolic polyamine oxidase (PAO) and mitochondrial monoamine oxidase (MAO). PAO participates in the interconversion metabolism of polyamines (PAs). MAOs are tightly bound to the outer mitochondrial membrane and occur in two isoforms, named A and B. In general, MAOs oxidize primary arylalkyl amines and are widely distributed among eukaryotes. In the present review, some physiopathological aspects of Cu-AO/histaminase (diamine oxidase, DAO) and the possible therapeutic applications of these enzymes will be discussed.

## Copper Amine Oxidases and Their Involvement in Physiology and Pathophysiology

Cu-AOs are dimeric proteins, each subunit weighing about 70–90 kDa, with 33 fully conserved residues near the catalytic site. The active site, consisting of one $Cu^{2+}$ and one TPQ connected by a water molecule, is located inside each subunit and is connected with the outer aqueous phase through a hydrophobic channel. In the last few years, the structure of several Cu-AOs has been revealed by means of X-ray chromatography and gene cloning[4]. The crystal structure of bovine serum AOs (BSAOs) was recently published[5].

A number of diverse physiological functions have been ascribed to AOs, although their exact role and biological activity are not completely understood. These enzymes are involved in different metabolic processes depending on the action of their substrates and reaction products. There is ample evidence that polyamines, $H_2O_2$, and aldehydes are of pivotal physiological relevance in cell proliferation, differentiation, and death[6]. For example, depending on its concentration, $H_2O_2$ can either impair cell growth and proliferation, or regulate gene expression and transduction of cell signaling[7]. Moreover, injection of as little as 0.3 units of pig kidney diamine oxidase (pkDAO), immobilized on concanavalin A-Sepharose, into the peritoneal cavity of Swiss mice 24 h after transplantation of neoplastic Ehrlich ascite cells, resulted in a strong inhibition of intraperitoneal tumor cell growth[8]. Of note, these cells contain a considerable amount of polyamine (PA), which appears to be oxidized by DAO. In this context, 99% inhibition of Ehrlich ascite cell proliferation was observed in the presence of oxidized spermine, whereas spermine had no effect[9]. Overall, these results may suggest a possible use of AOs in anticancer therapy[10]. As a matter of fact, BSAO, added to the incubation mixture of cultured Hamster fibroblasts in the presence of added polyamines, has a cytotoxic effect[11]. Cu-AOs may act by post-translational modification of proteins[12]; for instance, Cu-BSAO can mediate the oxidation of polypeptide amino groups, as occurs with synthetic polylysine, as well as endogenous lisozyme and ribonuclease A. At the cellular level, Cu-BSAO has been observed to modulate the electric properties of ion channels in neuroblastoma cells, thereby increasing the current of the $K^+$ channels[13]. At the organ level, Cu-BSAO was found to have a protective effect on cardiac function, as it exerts antiarrhythmic effects on isolated rat hearts subjected to regional ischemia and reperfusion[14]. This effect could be due, at least in part, to its antioxidant action against electrolysis-induced generation of oxygen free radicals and their by-products.

Cu-AOs are likely involved in leukocyte adhesion and extravasation. Salmi and Jalkanen[15] demonstrated that VAP-1, a cell surface endothelial glycoprotein, is actually a Cu-AO. This enzyme, which is sensitive to semicarbazide-specific inhibitors of AO is able to metabolize substrates like benzylamine and methylamine, and has striking molecular similarities to Cu-BSAO[16]. This enzyme exerts catalytic activity against plasma membrane–bound amino groups, such as the N-terminus of proteins, $NH_2$-containing amino acid residues, amino carbohydrates, etc. Overproduction of Cu-AOs appears to be dangerous, as observed in diabetes, heart attack, ischemia, and cataract. Cu-AO activity is increased in diabetes, particularly in diabetic patients with vascular complications, probably due to the production of endogenous cytotoxic compounds[17]. Transgenic diabetic mice showed an increase in Cu-AO activity in serum, kidney, and adipose tissue. This increased Cu-AO activity in diabetes is probably dependent on post-transcriptional modification or activation of prestored inactive enzyme molecules. These results, and in particular the role of diabetic vascular dysfunction on the negative effects of Cu-AO, are in agreement with the results of Ucar et al.[18]. They reported that elevated Cu-AO activity is correlated with $H_2O_2$ production in lungs with ischemia-reperfusion injury. Of note, in this model, ischemic preconditioning associated with Cu-AOs inhibition has a protective effect. In humans, enhancement of DAO activity has been directly related to cataract formation[19]. Lens proteins were modified by $H_2O_2$ production and/or post-translational modification due to Cu-AO activity[20]. In this case, FAD-MAO and PAO do not appear to be appreciably involved.

It should be pointed out that a proper concentration of Cu-AO and reactive oxygen species (ROS)–scavenging enzymes, such as superoxide dismutase and glutathione peroxidase, are crucial to explaining the beneficial/dangerous effects of copper Cu-AOs and in particular of histaminase. In this context, the identity of DAO and histaminase should be pointed out[21]. This enzyme shows a protective effect against various diseases. For example, it has been localized in decidual cells of human placenta[22], where it may be a local defense mechanism against noxious products of fetal origin, such as putrescine and PAs, which are produced in large amounts by tissues with a high proliferation rate[23]. Localization of DAO in kidney tubular cells suggests a role of DAO in regulating the concentration of histamine and other biogenic amines in blood and urine[24].

Other enzymes of the class of DAOs are the semicarbazide-sensitive AOs with affinity for benzylamine (Bz-SSAOs), or benzylamine oxidase, which are inhibited by carbonyl reagents and catalyze the oxidative deamination of several monoamines[25]. Benzylamine oxidase is a widely distributed enzyme, for it has been detected in various tissues of many animal species, including humans[26]; this enzyme can also contribute to histamine catabolism.

## Role of Diamine Oxidases in Anaphylaxis

Histamine plays a fundamental role in anaphylaxis, and histaminase is involved in allergic and pseudoallergic reactions. At variance with other metabolic pathways, histaminase activity is not directly up-regulated by endogenously released histamine. For instance, plasma histaminase activity increases in anaphylactic shock, but is not induced by released histamine, since it does not occur on histamine injection[27]. In some cases, histaminase levels may be intrinsically low and its activity further decreased by exogenous histamine, thereby predisposing to anaphylactic reactions. In a previous study, pigs were treated with 60 mg histamine. Half of the animals had histaminase inhibited by aminoguanidine and underwent marked elevation of plasma histamine (up to 160 μg/ml) associated with severe anaphylactic symptoms (e.g., hypotension, flushing, vomiting), causing some of them to die[28].

Enhanced histamine levels in humans may be related to many factors, endogenous or exogenous. Food-induced histaminosis was described by Sattler et al.[29] as the result of high histamine content or histamine releasers in food. The first symptom of excess histamine intake and/or release is increased gastric secretion, occurring at plasma histamine levels of about 2 μg/ml. Tachycardia and headache may appear at 5 μg/ml and hypotension at 6–8 μg/ml[30]. The amount of histamine in food varies widely, commonly ranging from 0.16 to 23 μg/g[31]. The largest amounts of histamine and tyramine, which have similar vasoactive properties, are found in fermented foods, such as cheese, red wine, tinned fish, fish sauces, *sauerkrauts*, tuna fish, cured pork, and sausages[32]. The histamine content of French cheeses may even reach values higher than 800 μg/g, thus causing toxic symptoms. High levels of histamine have been detected in Oriental food, accounting for the so-called Chinese restaurant syndrome[33]. Chemical *de novo* formation of amines may also occur during normal cooking and storage[34].

It should be pointed out that many commonly used drugs are DAO inhibitors and approximately 20% of the general population take these drugs[1]. Other factors (alcohol, spoiled food, etc.) can also block DAO activity. The concept of pseudoallergic reactions to food, or false food allergies, was first suggested by Dukor et al. in 1980[35]. The abnormal intake of biogenic amines like histamine and tyramine is one of the major mechanisms involved[30]. The main clinical symptoms are fall in blood pressure, angioedema, vasomotor headaches, defects of intestinal functions, cutaneous and respiratory symptoms, etc. Juhlin[36] suggests that authentic food allergy accounts for less than 3% of the cases of chronic urticaria, whereas false food allergies are involved in 40% of all cases. Abnormal intestinal permeability to histamine, either spontaneous or caused by irritant substances (e.g., alcohol, aspirin), could account for pseudoallergic reactions to food when symptoms arise after intake of abundant histamine-rich food. High levels of histamine may also derive from bacterial flora of the colon[37], especially in subjects with colonic dysmicrobism due to excessive intake of foods rich in cellulose and starch. Histamine of bacterial origin passes easily through the intestinal mucosa irritated by organic acids generated by fermentation[30].

Besides excess histamine, other histamine-dependent mechanisms of pseudoallergic reactions to food may include changes in the activity of histamine-degrading enzymes. A drop in histaminase and MAO B was found in patients with atopic dermatitis[38]. Histaminase may also be inhibited by food toxins, sodium nitrite, curarizing agents, antibiotics (i.e., clavulanic acid), and viral hepatitis[30,39,40]. Reduced DAO activity and histamine catabolism seem to be involved in bronchoconstriction episodes occurring after intake of red wine or histamine-rich food[41]. Of note, intestinal DAO is thought to be required for clearance of diamine and putrescine[42]. Overall, the above studies indicate enteral histaminosis resulting from dysfunctional metabolism of endogenous or exogenous histamine as a major cause of food intolerance[43].

Elevated mucosal histamine content and secretion were observed in the gut of patients with allergic enteropathy, as well as in women with Crohn's disease and ulcerative colitis[44,45]. In particular, evidence has been offered that mast cell–derived inflammatory mediators, including histamine, play a major pathogenic role in these diseases. Fogel et al.[46,47], using a model of ulcerative colitis in rats, demonstrated an overall improvement and reduction of the local inflammatory reaction after treatment with DAO from pig kidney immobilized on concanavalin A sepharose[46,47]. In particular, histopathologic changes in the colon of DAO-pretreated rats were markedly blunted, as were plasma ceruloplasmin activity and tissue myeloperoxidase[47]. These studies highlight the pivotal role of histamine in inflammatory bowel diseases, as well as the role of histaminase in controlling the levels of histamine in tissues and blood.

When considered altogether, the above findings support the concept of a possible therapeutic use of plant-derived histaminase, characterized by high specific activity and ability to degrade various biogenic amines, including histamine and polyamines, for allergic and pseudoallergic diseases, as better discussed below.

## Plant-Derived Diamine Oxidase

Plant DAO (Cu-AO; histaminase; EC 1.4.3.6) is physiologically involved in oxidative deamination of various biogenic amines to the corresponding amino-aldehydes. Production of $H_2O_2$ on amine degradation has been correlated with oxidative burst, cell death, as well as peroxidase-mediated lignification, suberization, and cell wall polymer cross-linking occurring during ontogenesis and defense responses[48,49]. DAO is the most abundant soluble protein detected in the extracellular fluids from *Fabaceae*, in particular, pea *(Pisum sativum)*, lentil *(Lens culinaris)*, and chickpea *(Cicer arietinum)* seedlings[50].

Plant DAOs (histaminase) differ from the mammalian and prokaryotic enzymes in a number of peculiar features, mainly high turnover rate of catalysis, high binding affinity for histamine, and high chemical stability. Moreover, this enzyme can be isolated to a high degree of purity with two simple and inexpensive chromatographic steps. Recently, an international Patent (n. PCT/EP01/13770) has been taken by some researchers of our group for a drug based on plant-derived histaminase for the treatment of histamine-mediated diseases, such as cardiac anaphylaxis, allergic asthma, allergic and septic shock, urticaria, rhinitis, and conjunctivitis. The main sources of histaminase are etiolated seedlings of leguminous plants, such as *P. sativum, L. culinaris, C. arietinum,* and *Latirus sativus,* where this enzyme is present in high concentrations, up to 4% of total protein content. Despite the promising pharmacological potential of plant DAOs, their actual therapeutic use is limited by their immunogenicity and short half-life in blood. To overcome these problems, purified wild pea DAO has been modified by binding to polyethylene glycol (PEG), a polymer that masks the protein surface, giving rise to a semi-synthetic derivative with decreased immunogenicity and catabolism, resulting in a prolonged half-life. In particular, animal studies have shown that PEGylation with a 20-kDa branched polymer totally eliminates DAO immunogenicity as judged by determination of IgM and IgG raised against the native enzyme, and causes a marked prolongation of detectable DAO levels in blood, thus markedly improving its pharmacokinetic profile. Of note, PEGylation did not interfere with DAO biological activity[51]. These findings offer good odds for the use of DAO/histaminase as a novel therapeutic approach to histamine-related disorders. To validate this assumption, it is useful to review the current studies on pea seedling histaminase applied to animal models of anaphylactic and inflammatory diseases.

## PLANT DIAMINE OXIDASE/HISTAMINASE MODULATES ALLERGIC ANAPHYLACTIC RESPONSE IN THE GUINEA PIG

The massive release of histamine from tissue mast cells elicited by the cross-linking of antigen with IgE bound to FCε receptors at the cell surface is considered the paramount event in type-I allergic reaction. The most commonly used experimental animal models of type-I allergic reaction encompass cardiac anaphylaxis *ex vivo* and asthma-like reaction in sensitized guinea pigs *in vivo*. Both these models have been used to investigate the therapeutic potential of pea seedling DAO.

### Protective Effect of Plant Histaminase on Cardiac Anaphylaxis

It is well known that histamine, one of the major mediators implicated in anaphylactic reactions, is released from heart slices and perfused hearts of sensitized guinea pigs on antigen challenge[52]. pkDAO has been demonstrated to have antihistaminic activity *in vivo* and a protective role in guinea pig anaphylactic shock[53]. Recently, we have investigated this issue by studying the effect of purified pea seedling histaminase[54], both free and immobilized on CNBr-Sepharose[55], in active cardiac anaphylaxis[56]. Briefly, guinea pigs were sensitized by repeated intraperitoneal injections of ovalbumin (OA) and, 18–25 days later, they were killed and the heart removed and perfused *ex vivo* in a Langendorff apparatus, which allows an accurate determination of the main cardiac functional parameters, namely heart rate, contraction strength, and coronary flow. Challenge with OA to the isolated hearts induced typical histamine-related changes in cardiac function. These changes were (1) transient increase followed by long-lasting reduction of myocardial contractility, (2) increased heart rate and occurrence of severe arrhythmias, (3) transient, marked reduction in the coronary flow followed by a less-pronounced, long-lasting phase of coronary constriction. In basal conditions, histamine content in the perfusates was low and, noteworthy, remained unchanged on addition of pea seedling histaminase. Histamine was released in the perfusates mostly during the first 5 min after OA challenge, whereas its levels returned close to the basal, prechallenge values after 30 min (Fig. 1). When OA challenge was performed in the presence of free or immobilized histaminase (4 IU/ml) the positive inotropic and chronotropic response to OA was fully blocked, including a dramatic decrease in the occurrence of ventricular tachyarrhythmias, and in the levels of histamine in the perfusates (Fig. 1).

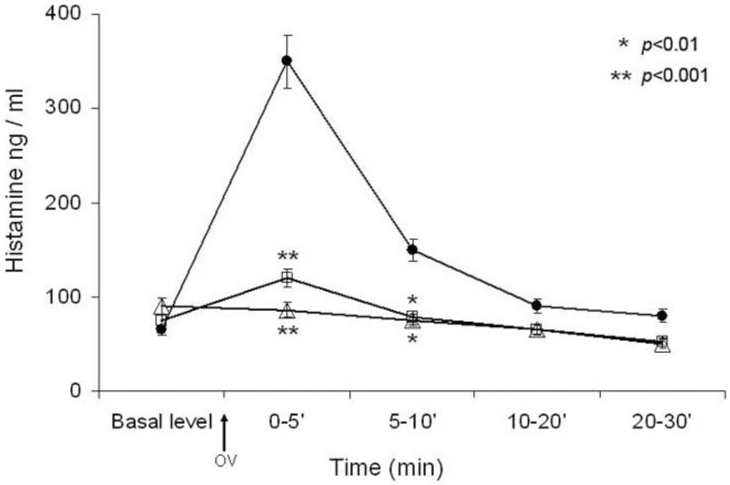

**FIGURE 1**. Histamine release in the perfusates from untreated control hearts (•) and from hearts perfused with free (□) or immobilized (Δ) histaminase (4 IU/ml). Data are expressed as mean ± SEM of eight experiments.

Cardiac mast cell granule content, evaluated by computer-aided densitometry on histological sections stained with a heparin-specific dye, was markedly decreased by OA challenge. As expected, none of the two assayed histaminase preparations reduced mast cell degranulation in response to the allergen.

The fact that histaminase does not produce any inhibitory, chromoglycate-like effect on anaphylactic degranulation of mast cells is strengthened by the finding that the depletion of cardiac histamine produced by OA was the same in the absence or presence of histaminase treatment. Altogether, these observations suggest that the observed decrease in the anaphylactic reaction of the isolated, perfused guinea pig hearts in response to OA is chiefly dependent on the inactivation of endogenously released histamine by histaminase, both free and immobilized.

It is well known that cyclic nucleotides and tissue $Ca^{2+}$ levels play a key role as intracellular effectors in type-I anaphylactic reactions[57]. In the current cardiac anaphylaxis model[56], OA challenge caused a slight decrease in cardiac cGMP and increase in tissue $Ca^{2+}$ levels, both effects prevented by histaminase treatment (Fig. 2A and B).

The mechanisms underlying these effects of histaminase on tissue cGMP and $Ca^{2+}$ remain a matter of speculation. Likely, this is not a direct effect of this enzyme, but may result from the interaction with the nitric oxide (NO) generation system, as NO can increase cardiac cGMP levels and decrease tissue $Ca^{2+}$ concentrations[58]. In fact, in control hearts, OA evoked a significant increase in the amount of nitrites, the stable oxidation products of NO, in the perfusates. It is notable that both free and immobilized histaminase significantly increased the amount of nitrites in the perfusates in the last phase of the anaphylactic reaction. Moreover, the two enzymes were able to significantly increase cardiac NO synthase activity[56].

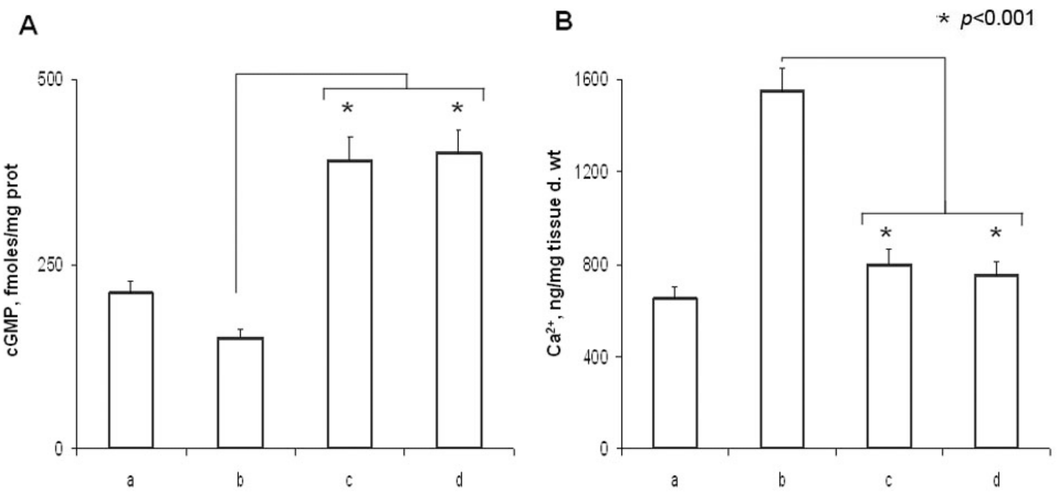

**FIGURE 2.** Tissue levels of cGMP (A) and calcium (B) in control hearts (a), hearts after OA challenge (b), and after OA challenge on perfusion with free (c) or immobilized (d) histaminase (4 IU/ml). Data are expressed as mean ± SEM of eight experiments.

## Protective Effect of Plant Histaminase on Allergic Asthma-Like Reaction

Allergic asthma is a major respiratory disease with a markedly increasing morbidity worldwide. Despite decades of research, its very complex, interwoven mechanisms are neither completely unveiled nor understood. Nevertheless, there is general agreement among basic and clinical scientists that histamine is a crucial mediator of inflammation and bronchospasm, two key features of allergic asthma[59]. On this ground, we carried out an experimental study to evaluate the effects of histaminase, free or immobilized, on asthma-like reaction induced in OA-sensitized guinea pigs by aerosolic exposure to the allergen. This animal model is known to reproduce respiratory abnormalities, airway hyper-responsiveness, and leukocyte lung infiltration resembling the functional and histopathologic hallmarks of human allergic asthma[60,61].

Histaminase (DAO, EC 1.4.3.6), free or immobilized on BrCN-Sepharose, 20 µg in 500 µl saline, was injected intraperitoneally and then tracked in blood, lung, and liver tissue to define its pharmacokinetic profile. Plasma histaminase levels were higher in the animals treated with free enzyme than in those treated with the immobilized one; as expected, no detectable enzyme was found in untreated animals. On the other hand, higher amounts of histaminase were found in lung and liver tissue from animals treated with the immobilized enzyme than in those treated with the free enzyme[60]. It was somewhat surprising that different amounts of histaminase were found in different organs and in blood on administration of the enzyme as free substance or BrCN-Sepharose–immobilized molecule. One possible explanation for the lower levels of immobilized histaminase in blood may be that BrCN-Sepharose used as the vehicle was not cross-linked, and thus tended to melt at body temperature. The unexpected, higher amounts of immobilized rather than free histaminase in lung and liver can be ascribed to the fact that homing of the enzyme conjugate in those organs may be facilitated by the presence of galactose units in the molecule of the Sepharose vehicle.

Challenge of sensitized guinea pigs with aerosolized OA resulted in severe abnormalities in their respiratory pattern, consisting of reduced latency time for the appearance of cough, increased cough severity, and increased occurrence of dyspnea and gasping (Fig. 3A and B), indicating the onset of respiratory failure. As expected, OA challenge caused no respiratory abnormalities in the nonsensitized guinea pigs used as negative controls. Pretreatment with free or immobilized histaminase, either given intraperitoneally at the noted dose of 20 µg i.p., or administered as aerosol solution, 4 µg/ml (flow rate 0.5 ml/min, corresponding to a total dose of 0.8 IU of the enzyme), resulted in a marked reduction of breathing

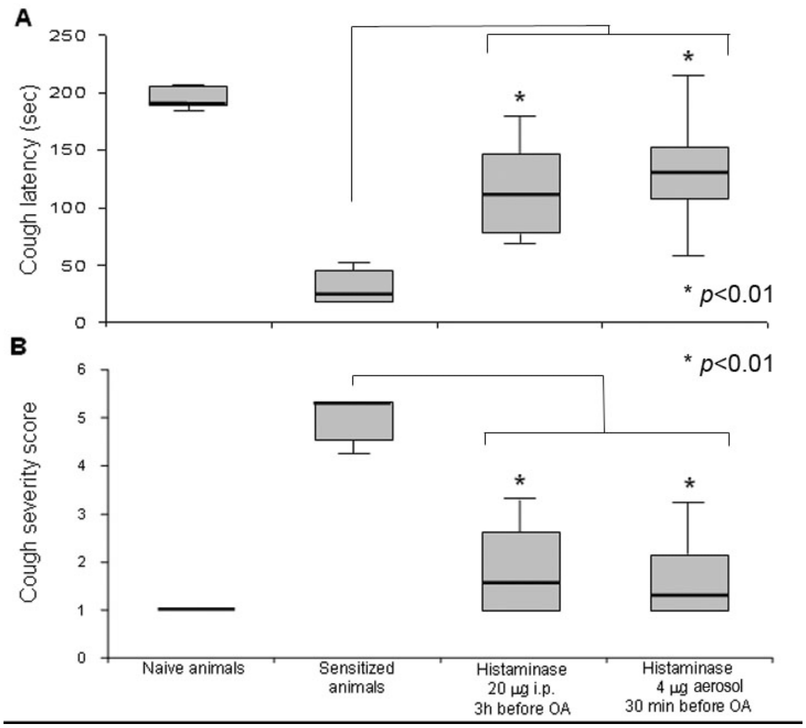

**FIGURE 3.** Effects of $CNB_2$-Sepharose 6MB immobilized histaminase (20 µg, given intraperitoneally 3 h before OA, or 4 µg/ml, given by aerosol 30 min before OA) on cough latency (A) and cough severity score (B) induced by OA challenge in sensitized guinea pigs. Data are expressed as box plot of 12 experiments; block lines represent median values, boxes represent interquartile range, whiskers represent extreme values.

abnormalities and prevention of respiratory failure (Fig. 3A and B). The maximal protective effects were observed when the enzyme was given by aerosol 30 min before or together with OA challenge (Fig. 3A and B)

Histaminase treatment also prevented the histopathological lung changes induced by OA challenge. Macroscopic examination of the lungs of the sensitized, OA-challenged guinea pigs showed marked swelling of the pulmonary lobes due to peripheral airway obstruction and air entrapment. Lung inflation was not observed on treatment with histaminase. Accordingly, light microscopic and morphometric analysis of lung tissue from the sensitized guinea pigs challenged with OA showed a marked, significant constriction of intrapulmonary bronchi and dilation of respiratory air spaces. On the other hand, histaminase treatment of the OA-challenged animals resulted in no appreciable signs of intrapulmonary bronchial constriction and respiratory air space dilation (Fig. 4 A–D).

As observed in the cardiac anaphylaxis model, mast cell granule content was markedly reduced in the sensitized guinea pigs challenged with OA, regardless of pretreatment with histaminase at any concentration and administration route, as compared with the nonsensitized negative controls. This finding further supports the notion that histaminase does not have any stabilizing, chromoglycate-like effect on allergen-induced granule release by lung mast cells.

Finally, histaminase treatment reduced lung inflammatory tissue injury, namely leukocyte infiltration and oxygen free radical generation. In fact, both free and immobilized histaminase markedly and significantly reduced myeloperoxidase (MPO) activity, an indication of tissue leukocyte infiltration, and malonyldialdehyde (MDA) production, an indication of peroxidation of cell membrane lipids by ROS, which were markedly increased in the lung tissue from sensitized, OA-challenged guinea pigs not given

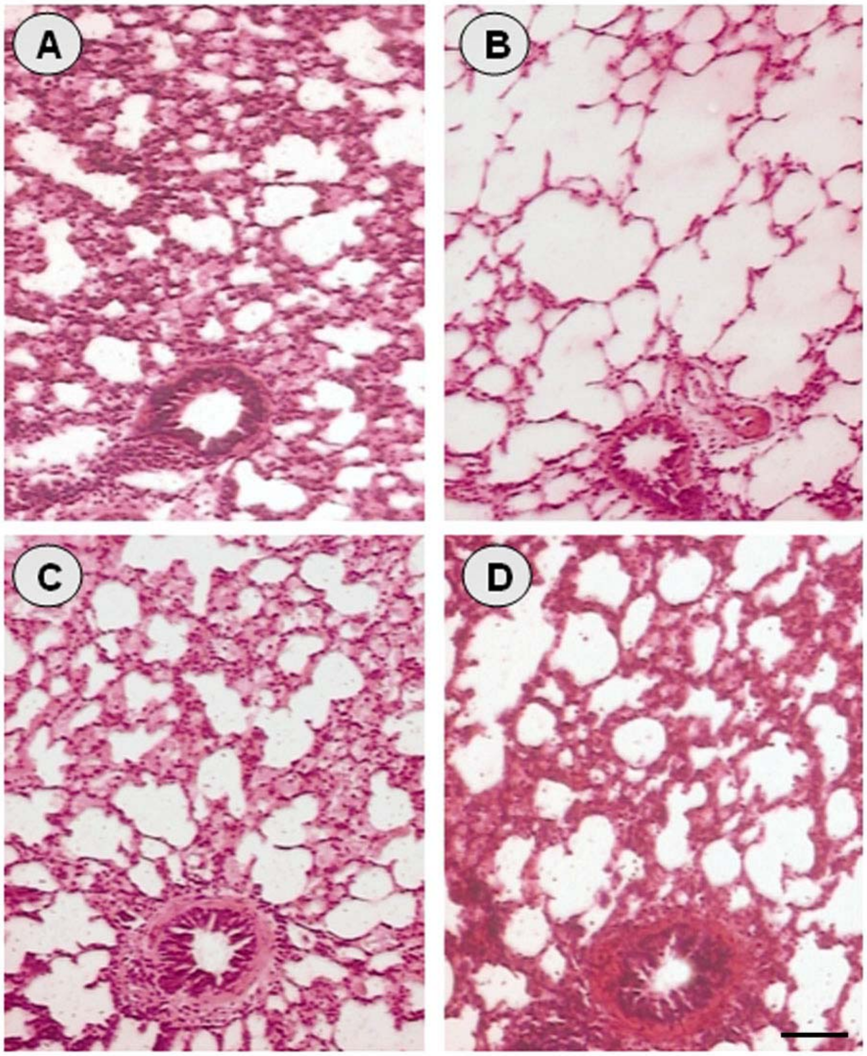

**FIGURE 4.** Representative lung tissue micrographs from (A) naïve guinea pig, (B) sensitized guinea pig undergoing OA aerosol, (C) sensitized guinea pig pretreated with aerosolic histaminase 30 min before OA aerosol, (D) sensitized guinea pig treated intraperitoneally with histaminase 3 h before OA aerosol. Hematoxylin & eosin; bar = 200 μm.

this enzyme compared to the unsensitized control animals. No difference in efficacy was noted between free and immobilized histaminase.

## PLANT DIAMINE OXIDASE/HISTAMINASE HAS A PROTECTIVE EFFECT AGAINST ISCHAEMIA-REPERFUSION INJURY

Besides its pivotal involvement in anaphylaxis and allergic reaction, histamine plays a role in exacerbation of inflammatory tissue damage, as many proinflammatory mediators, such as prostanoids and cytokines, are potent mast cell activators[62]. In particular, histamine has been found to contribute to endothelial dysfunction, hampering of blood-tissue exchange, and functional tissue impairment occurring in organs undergoing ischemia and reperfusion[63]. On these grounds, we hypothesized that pea seedling histaminase could effectively blunt the adverse effects of ischemia-reperfusion and we carried out experiments on specific animal models to prove this assumption. The results obtained have met our expectations, as summarized below.

## Protective Effect of Plant Histaminase in a Rat *In Vivo* Model of Cardiac Ischemia-Reperfusion

The heart is particularly susceptible to tissue damage by several key agents involved in the pathophysiological mechanisms of ischemia-reperfusion, such as ROS, histamine, and NO[64,65,66]. A close relationship between histamine, acting as a pro-oxidant, and ROS was observed in patients with coronary heart disease[67]. In turn, histamine release by mast cells is amplified by excess superoxide generation and concurrent decrease in local NO amounts, due to the fact that superoxide and NO react promptly, giving rise to harmful peroxynitrite[68]. Moreover, histamine *per se* may induce a spasm of the coronary vessels, thereby causing or worsening acute coronary insufficiency and myocardial ischemia[63]. During ischemia, the increase in histamine levels in the coronary sinus is paralleled by the occurrence and severity of ventricular arrhythmias[69]. To assess whether pea seedling histaminase has cardioprotective effects against postischemic reperfusion damage, we used a typical *in vivo* model in the rat. Briefly, anesthetized, artificially ventilated rats were subjected to transient ischemia (30 min) by temporary occlusion of the left anterior descending coronary artery, followed by reperfusion (60 min) by removal of the arterial occlusion. Sham-operated rats were the negative controls. This model allows for continuous ECG monitoring of the animals and, at the end of reperfusion, appropriate cardiac tissue sampling for pharmacological and histopathological assays[70]. Some animals from each experimental group were excluded from tissue sampling and used to evaluate the area at risk, i.e., the cardiac area rendered ischemic by the coronary occlusion that remains unstained after an intracoronary bolus of methylene blue, and the infarcted area, i.e., the nonviable myocardial tissue area downstream of the coronary occlusion that remains unstained on incubation with the vital dye nitroblue tetrazolium[70].

Histaminase (DAO, EC 1.4.3.6), at the dose of 80 IU/kg b.w., given intravenously either 10 min before reperfusion or exactly at reperfusion, reduces the size of myocardial infarction caused by ischemia-reperfusion. By morphometrical evaluation, the area at risk was similar in the rats subjected to ischemia-reperfusion treated or not with histaminase, while the infarcted area, expressed as percent of the corresponding area at risk, was significantly reduced on histaminase administration (Fig. 5). No cardiac protection was afforded by semicarbazide-inactivated histaminase[70] replaced by the bioactive enzyme.

Analysis of ECG recordings showed that histaminase reduced the occurrence of ventricular arrhythmias. Overall, the number of animals which survived the 60-min reperfusion was higher in the histaminase-treated groups than in the untreated one. Histaminase also reduced the biochemical tissue alterations induced by ischemia-reperfusion. In particular, MDA production (an index for peroxidation of cell membrane lipids by ROS), MPO activity (an index for tissue leukocyte infiltration), and calcium content, which were significantly elevated in the rats subjected to ischemia-reperfusion as compared with the sham-operated ones, were reduced by histaminase treatment. Moreover, histaminase prevented the activation of caspase 3, the initiator enzyme of the apoptotic cascade, induced by ischemia-reperfusion.

In conclusion, plant histaminase appears to protect the heart from the deleterious effects of ischemia-reperfusion[70]. The mechanisms of this effect mainly rely on histamine catabolism and removal of the proinflammatory and arrhythmogenic actions of this biogenic amine. In addition, histaminase may also act through its ROS scavenging properties. Overall, histaminase administration may offer good prospects for the development of new drugs to reduce the adverse effects of reperfusion in patients with acute coronary syndrome subjected to catheter-based angioplasty.

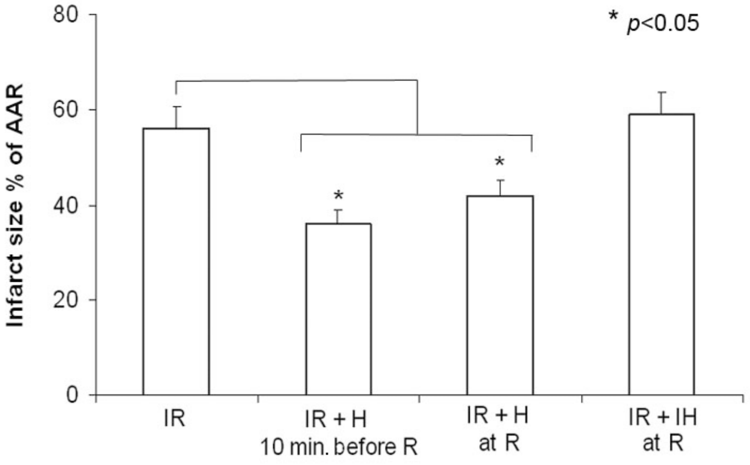

**FIGURE 5.** Myocardial infarction size after 30-min ischemia and 60-min reperfusion (IR) expressed as percent of the area-at-risk (AAR), evaluated by computer-assisted morphometry on nitroblue tetrazolium–stained hearts. H: histaminase (80 IU/kg b.w.), IH: semicarbazide-inactivated histaminase (same dose). Results are expressed as mean ± SEM of eight experiments.

## Protective Effect of Plant Histaminase in a Rat *In Vivo* Model of Splanchnic Ischemia-Reperfusion-Induced Shock

Splanchnic ischemia may result from different causes, leading to general or local hypoperfusion and impaired blood supply to the bowel[71]. Consequences of splanchnic ischemia range from persistent intestinal bleeding to bowel perforation and peritonitis. Thus, surgical resection of the affected bowel segment is usually required to minimize adverse outcomes[72]. The pathophysiology of intestinal ischemia has been widely investigated in animal models, mainly based on surgical occlusion of the splanchnic circulation followed by reperfusion (splanchnic artery occlusion/reperfusion, SAO/R). This results in intestinal injury and circulatory shock, with a high mortality rate[73,74]. These models permit investigation of the pathophysiology of SAO/R, which primarily involves endothelial dysfunction and leukocyte recruitment and activation, eventually leading to inflammation-mediated tissue injury. Histamine secretion from inflammation-activated intestinal mast cells greatly contributes to lethal circulatory shock occurring on SAO/R. Histamine is released by the intestinal mucosa mostly during reperfusion[75] and sparks a vicious cycle that leads to further endothelial activation and leukocyte adhesion and extravasation[76]. We have therefore investigated the therapeutic effects of pea seedling histaminase in a rodent model of SAO/R and circulatory shock. Anesthetized rats underwent a temporary (45 min) clamping of the celiac and superior mesenteric arteries near their aortic origins. Subsequent reperfusion was monitored for up to 6 h. Blood pressure was registered throughout the experiment. Sham-operated animals not subjected to arterial clamping were the negative controls[77].

Histaminase, 80 IU/kg b.w./0.5 ml of phosphate buffer, administered 15 min before reperfusion, significantly reduced the drop in blood pressure and high mortality rate caused by SAO/R. Histaminase also reduced histopathological changes, leukocyte infiltration (MPO activity), and expression of endothelial cell adhesion molecules in the ileum[77]. Besides reducing local tissue inflammation through acceleration of histamine catabolism, histaminase also seemed to counteract ROS-mediated tissue injury, as judged by the significant decrease in the tissue levels of peroxidation and nitration products (oxidized rhodamine, MDA, and nitrotyrosine), DNA damage markers (8-hydroxy-2’-deoxyguanosine and poly-ADP-ribosylated DNA), and by consumption of tissue antioxidant enzymes, such as superoxide dismutase. As a result, histaminase led to a reduction in ileal cell apoptosis, as assessed by analysis of caspase 3 activity and of the number of TUNEL-positive cells.

In keeping with the findings from cardiac ischemia-reperfusion, the present study offers evidence that histaminase can afford protection against SAO/R-induced splanchnic injury, likely due to oxidative catabolism of proinflammatory histamine as well as antioxidant effects[78], resulting in hindrance of free radical–mediated tissue injury endothelial dysfunction and leukocyte recruitment[77]. Based on the above, and given that no definite medical therapy for intestinal ischemia currently exists, histaminase may be a possible candidate for the treatment of this disease.

## CONCLUSIONS AND PERSPECTIVES

Histamine and other primary amines released during anaphylactic reactions undergo oxidative deamination by histaminase. A series of pharmacological studies from our group indicate that pea seedling histaminase counteracts cardiac anaphylaxis and pulmonary asthma-like reaction to aerosolized allergen in sensitized guinea pigs. The mechanisms by which histaminase exerts its beneficial action appear to consist mainly in the inactivation of endogenously released histamine and, secondly, in an antioxidant, ROS-scavenging action. In addition to histamine, ROS also play a key pathogenic role in allergic asthma and other inflammatory lung diseases, as they are harmful and responsible for cell dysfunction, damage, and death. Moreover, histamine and pro-oxidant species, e.g., superoxide anion, massively released on allergen challenge, can account for bronchospasm, vascular edema, and leukocyte recruitment. In our studies, treatment with pea seedling histaminase markedly decreased the adverse effects of cardiac and lung anaphylaxis due to allergen challenge. The absence of any direct effect on mast cell granule release suggests that none of the tested histaminase preparations, both free and immobilized on BrCN-Sepharose, produce any cytoprotective chromoglycate-like effect on mast cells.

In the above animal models, histaminase treatment strongly decreased the tissue levels of MDA, nitrotyrosine, and the markers of oxidative DNA damage, strongly suggesting that this enzyme can reduce oxidative tissue damage induced by ROS generated during the anaphylactic reaction. This antioxidant-like protection of plant histaminase fits well with recent data, showing that BSAO exerts an *in vitro* antioxidant effect against electrolysis-induced oxidative species and *ex vivo* cardioprotective action against oxidative injury on isolated rat heart, comparable to the cardioprotective action of authentic antioxidant drugs, such as mannitol or desferoxamine[78,79]. On the other hand, it should be also taken into account that monoamine degradation by MAO can lead to the release of ROS, which in turn are involved in endothelial dysfunction, vasoconstriction, and tissue damage. In this context, paradoxically, histaminase on one hand and MAO-A inhibitors on the other hand can have therapeutic potential, as recently shown in studies in which blockade of 5-HT metabolism by pargyline, a MAO-A, inhibitor, affords protection against cardiac ischemia-reperfusion[80].

In conclusion, the results of the studies summarized in this review highlight the therapeutic potential of plant-derived histaminase in allergic diseases and anaphylaxis, as well as its ability to inhibit inflammatory and ROS-mediated tissue injury due to ischemia-reperfusion. Recent progress with polymeric conjugates of histaminase should enhance the pharmacological profile of this enzyme, enabling it to be administered by alternative routes, such as aerosol, which appear to be as effective as the parenteral ones.

## ACKNOWLEDGMENTS

This research was supported by grants from Ente Cassa di Risparmio di Firenze, Italy. The authors are grateful to Mrs. Mary Forrest for English language review.
